# Reflectance confocal microscopy features of chronic radiodermatitis: A useful tool for presurgical mapping

**DOI:** 10.1111/srt.13621

**Published:** 2024-02-23

**Authors:** Luca Rapparini, Federico Venturi, Chiara Gelati, Federico Giorgini, Marco Pignatti, Michelangelo La Placa, Biagio Scotti, Giulia Veronesi, Emi Dika

**Affiliations:** ^1^ Oncologic Dermatology Unit IRCCS Azienda Ospedaliero‐Universitaria di Bologna Bologna Italy; ^2^ Department of Medical and Surgical Sciences Alma Mater Studiorum University of Bologna Bologna Italy; ^3^ Plastic Surgery IRCCS Azienda Ospedaliero‐Universitaria di Bologna Bologna Italy

Dear Editor,

Reflectance confocal microscopy (RCM) is a useful tool for the diagnosis of skin cancers and inflammatory disorders.^1^, ^2^, ^3^, ^4^, ^5^ We present the case of a 75‐year‐old woman with a history of infiltrating lobular right breast cancer (pT2N0M0, UICC TNM 5th edition), treated with radical mastectomy, cobalt radiotherapy and breast reconstruction with an abdominal flap in 1997. Recent mammography and CT scan were negative for tumour recurrence. In October 2023, the patient was referred to our unit for the evaluation of an erythematous plaque located in the right mammary area. At clinical examination, an erythematous macule surmounted by a large crusted, fibrinous plaque was observed (Figure 1A). Dermoscopic examination of the lesion revealed an atypical vascular pattern at the periphery of the central keratin mass (Figure 1B). The whole skin appeared intensely photodamaged. Two incisional biopsies of the ulcerated plaque were performed. Histopathological evaluation led to the diagnosis of a post‐radiotherapy atypical vascular lesion (sec. WHO 2020). We further performed RCM for presurgical mapping of the lesion, which revealed, at the level of the stratum corneum, a severely disrupted corneal layer with presence of parakeratoses and detached corneocytes, whereas at the level of the stratum granulosum areas of necrosis corresponding to dark spaces with irregular demarcation and pronounced disruption of the epidermal architecture. Surrounding keratinocytes displayed the presence of spongiosis, seen as increased brightness of intercellular spaces, accentuating the honeycombed pattern of the granular layer. RCM changes observed were compatible with chronic radiodermitis (Figure 2A). Due to the presence of severe photodamage, RCM was performed contralaterally on the left mammary skin, and displayed an irregular epidermal honeycomb pattern with curled collagen in the dermis (Figure 2B). Subsequently, the patient underwent complete surgical excision of the atypical vascular lesion confirming the histopathologic analysis of the first incisional biopsies. Post‐radiation vascular lesions (including angiosarcoma and atypical vascular lesions) are a rare complication of irradiated skin in breast cancer patients. The latter present clinically as solitary or multiple reddish‐brown papules and enter the differential diagnosis with breast cancer recurrence, post‐traumatic haemorrhage, and benign dermatological conditions. They rarely progress to angiosarcoma, so the treatment of choice is surgical excision and follow‐up. In the literature, RCM has been used for the evaluation of acute radiation dermatitis toxicity, but there are no described cases of the application in chronic radiodermatitis. In acute radiation dermatitis histopathologic changes were detectable by RCM after a mean time of 15 days, compared with 30 days for clinical manifestations, and include spongiosis, exocytosis and inflammatory cells, dendritic‐shaped cells, streaming‐like figures, broken geographic papillae, epidermal architectural disarray, effacement of rete ridges, melanophages and hyperpigmentation of the basal layer^5^. Conversely, from what can be deduced from RCM examination in our case, the characteristics of chronic radiodermatitis with concomitant post‐radiation atypical vascular lesion are severe parakeratoses and detached corneocytes, together with areas of necrosis and spongiosis. To date, this is the first report of RCM features of chronic radiodermatitis. Further studies with larger samples are needed to clearly define RCM presentation of such entity.

**FIGURE 1:**
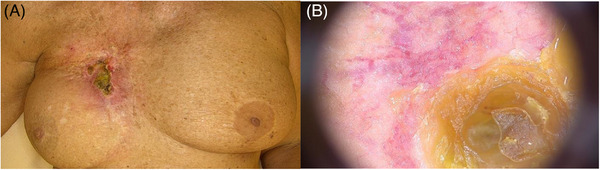
Clinical and dermoscopic presentation of radiodermatitis: clinical examination showed an erythematous macule surmounted by a large crusted, fibrinous plaque (A). Dermoscopy revealed an atypical vascular pattern characterized by large vessels at the periphery of the central keratin mass (B).

**FIGURE 2:**
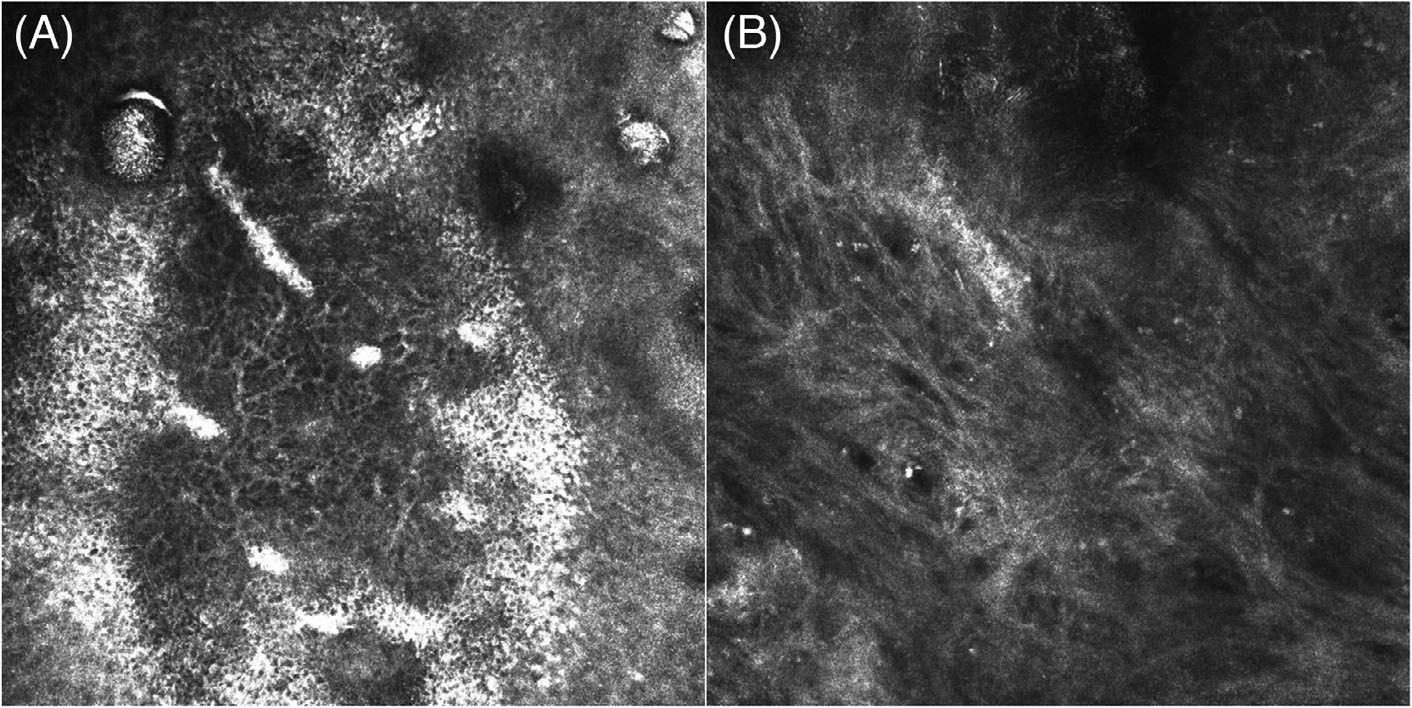
RCM features of radiodermitis revealed, at the level of the stratum corneum, a severely disrupted corneal layer with presence of parakeratoses and detached corneocytes, whereas at the level of the stratum granulosum we observed areas of necrosis corresponding to dark spaces with irregular demarcation and pronounced disruption of the epidermal architecture. Surrounding keratinocytes displayed the presence of spongiosis, seen as increased brightness of intercellular spaces, accentuating the honeycombed pattern of the granular layer (A). Due to the presence of severe photodamage, RCM was performed also contralaterally on the left mammary skin, which displayed an irregular epidermal honeycomb pattern with curled collagen in the dermis (B).

## CONFLICTS OF INTEREST STATEMENT

The authors have no relevant financial or non‐financial interests to disclose.

## Data Availability

Data sharing not applicable to this article as no datasets were generated or analysed during the current study.
